# Responsive materials and mechanisms as thermal safety systems for skin-interfaced electronic devices

**DOI:** 10.1038/s41467-023-36690-y

**Published:** 2023-02-23

**Authors:** Seonggwang Yoo, Tianyu Yang, Minsu Park, Hyoyoung Jeong, Young Joong Lee, Donghwi Cho, Joohee Kim, Sung Soo Kwak, Jaeho Shin, Yoonseok Park, Yue Wang, Nenad Miljkovic, William P. King, John A. Rogers

**Affiliations:** 1 Querrey Simpson Institute for Bioelectronics, Northwestern University, Evanston, IL 60208 USA; 2 Department of Mechanical Science and Engineering, University of Illinois at Urbana-Champaign, Urbana, IL 61801 USA; 3 Department of Electrical and Computer Engineering, University of California, Davis, CA 95616 USA; 4 Thin Film Materials Research Center, Korea Research Institute of Chemical Technology, Daejeon, 34114 Republic of Korea; 5 Bionics Research Center of Biomedical Research Division, Korea Institute of Science and Technology, Seoul, 02792 Republic of Korea; 6 Department of Advanced Materials Engineering for Information and Electronics, Kyung Hee University, Yongin, 17104 Republic of Korea; 7 Department of Biomedical Engineering, Northwestern University, Evanston, IL 60208 USA; 8 Department of Materials Science and Engineering, Northwestern University, Evanston, IL 60208 USA; 9 Department of Chemistry, Northwestern University, Evanston, IL 60208 USA; 10 Department of Neurological Surgery, Feinberg School of Medicine, Northwestern University, Chicago, IL 60611 USA

**Keywords:** Electronic devices, Health care

## Abstract

Soft, wireless physiological sensors that gently adhere to the skin are capable of continuous clinical-grade health monitoring in hospital and/or home settings, of particular value to critically ill infants and other vulnerable patients, but they present risks for injury upon thermal failure. This paper introduces an active materials approach that automatically minimizes such risks, to complement traditional schemes that rely on integrated sensors and electronic control circuits. The strategy exploits thin, flexible bladders that contain small volumes of liquid with boiling points a few degrees above body temperature. When the heat exceeds the safe range, vaporization rapidly forms highly effective, thermally insulating structures and delaminates the device from the skin, thereby eliminating any danger to the skin. Experimental and computational thermomechanical studies and demonstrations in a skin-interfaced mechano-acoustic sensor illustrate the effectiveness of this simple thermal safety system and suggest its applicability to nearly any class of skin-integrated device technology.

## Abstract (web summary)

Minimizing patient risk to thermal failure in wearable electronics typically requires complex circuit control, sensors, or passive materials. Here, Yoo et al. present an active materials system with a self-inflating bladder that delaminates upon excessive heat, mitigating the risk of thermal injury.

## Introduction

The value of continuous health monitoring in hospital and home settings motivates research on soft, wireless, skin-interfaced electronic devices as replacements for traditional wired sensors that require separate data acquisition electronics^1–3^. For patients such as critically ill infants, these technologies represent transformative improvements that bypass myriad difficulties associated with conventional monitoring equipment^4,5^. As with any healthcare technology, minimizing risks to the patient represents an essential goal in engineering design. Examples of such risks for wireless monitors include potential adverse reactions^5,6^ to the skin adhesives and, for infants, the possibility of choking by accidental ingestion of miniaturized devices^7^. Careful choices of materials serve as the basis for mitigating both of these risks. Another concern is in thermal injuries to the skin due to excessive heat generation in the electronics and/or the batteries^8,9^. The standard approach to address this possibility relies on sensors and associated electronic control logic^10–13^. An automated, materials-based scheme could provide an additional layer of protection. Passive materials with low thermal conductivity^14,15^, can be integrated into the soft encapsulating structures to provide some level of thermal isolation, although limited by simultaneous requirements for thin, flexible geometries that can support soft, comfortable interfaces to the curved surfaces of the skin. Advanced approaches exploit heat-absorbing phase transition materials^16,17^, but with similar constraints.

This paper presents an active materials system that avoids these limitations. The construct relies on a thin, thermally triggered actuator located in the encapsulating structure of the device, positioned adjacent to the surface of the skin at locations that represent the greatest risk for thermal injury. The actuator incorporates a small volume of liquid with a boiling point slightly above body temperature, enclosed in a thin, inflatable bladder. Vaporization occurs at temperatures slightly below those that could be harmful to the skin, thereby automatically inflating the bladder to establish a highly efficient thermal barrier and to actively delaminate the device from the skin. These two processes effectively eliminate the risk of thermal injury, with negligible effect on the thickness, weight, flexibility, size, cost, power consumption or complexity of the device, compared to passive materials and electronic components and circuit designs. Experimental measurements and finite element analysis of simulated thermal runaways in rechargeable batteries reveal the key considerations in choices of materials and engineering designs. Demonstrations with a wireless mechano-acoustic sensor during a catastrophic failure of the battery illustrate the practical aspects and efficacy of this system.

## Results

### Representative use case and design approach

**Fig. 1:**
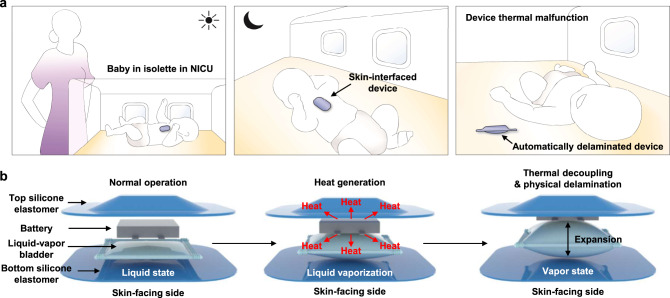
Automatic thermal safety system to protect the skin from overheating of a skin-interfaced, wireless electronic device. **a** Illustrations of a baby in an isolette in a NICU with a device mounted on the chest. Heating associated with device malfunction automatically activates the safety system to thermally isolate and physically delaminate the device from the skin to avoid injury. **b** Operating mechanism of this system, based on expansion of a bladder. The heat generated by the device transfers to the bladder, thereby triggering liquid vaporization. Expansion of the bladder removes the source of heat from the skin.

Figure 1a illustrates a use scenario that involves a skin-interfaced device for wireless monitoring of the vital signs of an infant in an isolette in a neonatal intensive care unit (NICU). This miniature device contains a small battery along with sensors, analog front end electronics and a radio communications module, to eliminate wires and other burdens associated with traditional monitoring equipment for this class of patients. Thermal risks to the skin associated with rapid discharge of the battery and/or excessive power dissipation in the active components of the system are typically addressed by use of electronic sensors and closed-loop integrated circuits that immediately terminate operation upon overheating. Devices that undergo any form of thermal malfunction must be immediately removed from the skin and permanently discarded. A drawback of these approaches is that these engineered safety systems themselves could fail to operate properly. As a result, schemes that rely on intrinsic properties of active materials are attractive, as complementary or alternative methods to enhance the thermal safety of skin-interfaced electronic devices, as they eliminate the complexity and the potential for failure of traditional schemes that rely on electronic components and circuit designs. The concept introduced here relies on a thermally triggered and self-powered mechanism that integrates directly into the soft, elastomeric encapsulating structure of the device itself. Operation thermally and mechanically decouples the device from the skin in a rapid manner in the event of an increase in temperature from a low, skin-safe threshold value. The mechanisms provide protection against thermal skin injury, continuously and in a mode that does not require a power supply.

As shown in Fig. 1b, the system relies on a thin, flexible bladder that contains a low boiling point liquid (Novec 71DE, a 50/50 (weight) mixture of C_5_H_3_OF_9_, 2-(difluoromethoxymethyl)−1,1,1,2,3,3,3,heptafluoro-propane and t-DCE (C_2_H_2_Cl_2_, trans-1,2-dichloroethylene), boiling point = 41 °C)^18^, positioned between the battery and the skin, inside the elastomeric enclosure of the completed device. For the example reported here, the membrane that defines this bladder has a thickness of 100 μm, with lateral dimensions of 20 mm × 20 mm. The total volume of liquid is ~10 μL, corresponding to a thickness of ~25 μm. As a result, the total thickness of the system is only ~125 μm, the weight is ~40 mg, and the flexural rigidity is only 1.7 ~ 5.0 nN·m^2^, thereby allowing it to be integrated into the device with negligible change in overall size, weight, or mechanical properties.

In normal operation, the temperatures of the device and the skin remain below the boiling point of the liquid. Temperatures that rise above the boiling point vaporize the liquid, thereby activating the thermal protection mechanism. Specifically, the vapor produced in this manner expands the bladder from a thickness of 125 μm at room temperature to nearly 5 mm at 46.6 °C, thereby delaminating the device from the skin and, simultaneously, forming a highly effective thermal barrier that isolates the heat source from the skin. (Supplementary Figs. 1, 2).

### Heat-triggered thermomechanical decoupling

**Fig. 2:**
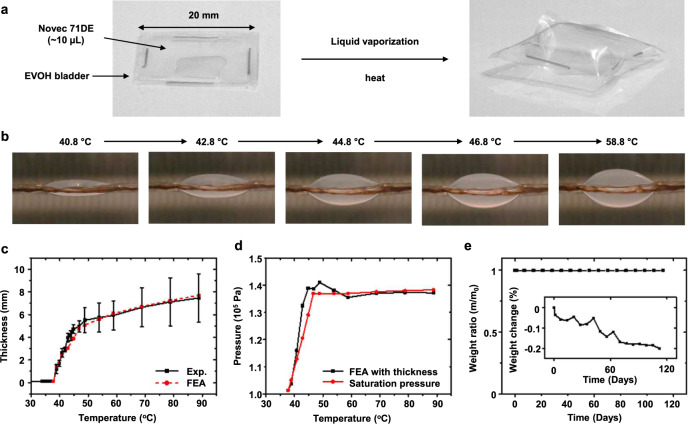
Thermomechanical expansion of a liquid–vapor bladder. **a** Photographs of liquid–vapor bladders before and after heating. The heat vaporizes the liquid and expands the bladder. The lateral dimensions of the bladder are 20 mm by 20 mm, and the volume of liquid (Novec 71DE) enclosed is ~10 µL. **b** Photographs of liquid–vapor bladders expanded at different temperatures. **c** Maximum thickness as a function of temperature for expanded liquid–vapor bladders loaded with ~10 μL volumes of liquid. Solid and dashed lines represent results based on experiments and FEA simulations, respectively. These data correspond to bladders held at each temperature for 20 min, to ensure steady state behavior. **d** Estimated inner pressure of the bladder corresponding to its maximum thickness by FEA simulation (black), and intrinsic saturation pressure of liquid inside the bladder (red). **e** Weight ratio (m/m_0_) and relative change (%, inset) of Novec 71DE liquid inside the EVOH bladder as a function of time at room temperature.

Figure 2a shows photographs of the bladder filled with a small volume (~10 μL) of Novec 71DE, non-flammable liquid with a low boiling point at atmospheric conditions (41 °C at 1 atm) and a low toxicity. A bladder made of a thin, transparent film (Non-catch, 50 μm, Kyodoprinting) mainly composed of ethylene vinyl alcohol (EVOH) contains the liquid and its vapor, without leakage^19^. Sealing of the EVOH films occurs by heat pressing, using methods that allow mass production of bladders at low cost. In addition, the sealed bladder contains the liquid even after the cyclic bending (~1 mm, 1000 cycles), and does not fracture until ~340% of strain (Supplementary Figs. 3, 4). The details appear in the Methods.

As mentioned above, for temperatures below 41 °C, such as those associated with device storage at room temperature (~25 °C) or with continuous operation on the surface of the skin (~35 °C), the Novec 71DE remains in a liquid state, and the bladder is correspondingly thin. Increases in temperature above 41 °C caused by device overheating or other circumstances lead to rapid evaporation of the Novec 71DE and to a resulting increase in pressure that dramatically expands the bladder, as illustrated in Fig. 2b and Supplementary Fig. 5. These demonstration results use an external heat source from an oven to increase the temperature in increments of 1 °C for 20 min each to ensure steady state in temperature, the extent of vaporization and the degree of expansion. After insertion into an oven at 48 °C, bladder starts to expand at ~10 s (Supplementary Fig. 6). The expansion reaches 50% of its maximum state after 30 s; complete expansion occurs after 1 min (Supplementary Fig. 6). For these studies, full vaporization of ~10 μL of Novec 71DE occurs at 46.6 °C and further increases in temperature lead to additional increases in the volume of the bladder due to thermal expansion of the vapor and the thermal plastic bladder (Fig. 2c and Supplementary Fig. 7, 8).

The bladder and its corresponding state of expansion strongly influence the thermal resistance between the device and the skin, *R*_bladder_, according to

1 $${R}_{{{{{{\rm{bladder}}}}}}}	={t}_{{{{{{\rm{vapor}}}}}}}/{k}_{{{{{{\rm{vapor}}}}}}}+{t}_{{{{{{\rm{liquid}}}}}}}/{k}_{{{{{{\rm{liquid}}}}}}}\\ 	={t}_{{{{{{\rm{bladder}}}}}}}/{k}_{{{{{{\rm{vapor}}}}}}}-{t}_{{{{{{\rm{liquid}}}}}}}(1/{k}_{{{{{{\rm{vapor}}}}}}}-1/{k}_{{{{{{\rm{liquid}}}}}}})$$

where *t*_bladder_ = *t*_vapor_ + *t*_liquid_ is the total thickness of the bladder due to contributions of the Novec 71DE in the liquid (*t*_liquid_) and vapor (*t*_vapor_) states; the corresponding thermal conductivities are *k*_liquid_ and *k*_vapor_, respectively. The thermal resistances of the bladder membranes are negligible because of their small thicknesses and relatively high thermal conductivity (0.35 W/(m · K))^20^. Consistent with expectation, this equation suggests that maximizing *t*_bladder_ and minimizing *t*_liquid_ maximizes the thermal resistance, and therefore the thermal barrier performance of this structure. For safe operation on the skin, the temperature can reach 43 °C for over 10 min, or 48 °C for 1–10 min, according to medical standards^21^. When the temperature is 46.8 °C, the experimentally measured maximum thicknesses of bladders filled with ~10 μL of Novec 71DE are 4.9 ± 0.8 mm. The corresponding result from three dimensional (3D) finite element analysis (FEA) is 4.71 mm (Fig. 2c). Furthermore, these simulations indicate that the inner pressure of the bladder is ~138 kPa, comparable to the intrinsic liquid–vapor saturation pressure (136 kPa), consistent with a negligible residual amount of liquid in the bladder at this temperature (Fig. 2d). For temperatures >46.6 °C, 10 μL of Novec liquid inside 20 mm × 20 mm EVOH bladder fully vaporizes (Supplementary Fig. 9), such that no liquid remains and the inner pressure is simply the vapor pressure that stabilizes the bladder expansion. Volumes <10 μL lead to depletion of liquid at reduced temperatures, resulting in lower saturation pressures and less bladder expansion. Volumes more than 10 μL achieve depletion at temperatures above 48 °C, allowing a further increase in bladder temperature before reaching a fully vaporized bladder. The design of the thermal safety system maintains the temperature at the skin interface to values below the medical standard (48 °C for 1–10 min exposure). A 10 μL volume of Novec 71DE inside a 20 mm × 20 mm EVOH bladder fully evaporates at 46.6 °C (Supplementary Fig. 9), slightly below the 48 °C. Since the fracture strains of the EVOH films are in the range of 130–330%^20^, the bladders can expand to large volumes, while maintaining a robust containment structure for inner pressures up to 174 kPa. These films are also highly impermeable; over a period of ~100 days at room temperature, the weight of the contained Novec liquid decreases by <0.2% (Fig. 2e). The expansion of these bladders proceeds with negligible effect of the overlying weight of the device components, including the battery (generally below 10 g) (Supplementary Fig. 10).

### Responses to simulated battery failures

**Fig. 3:**
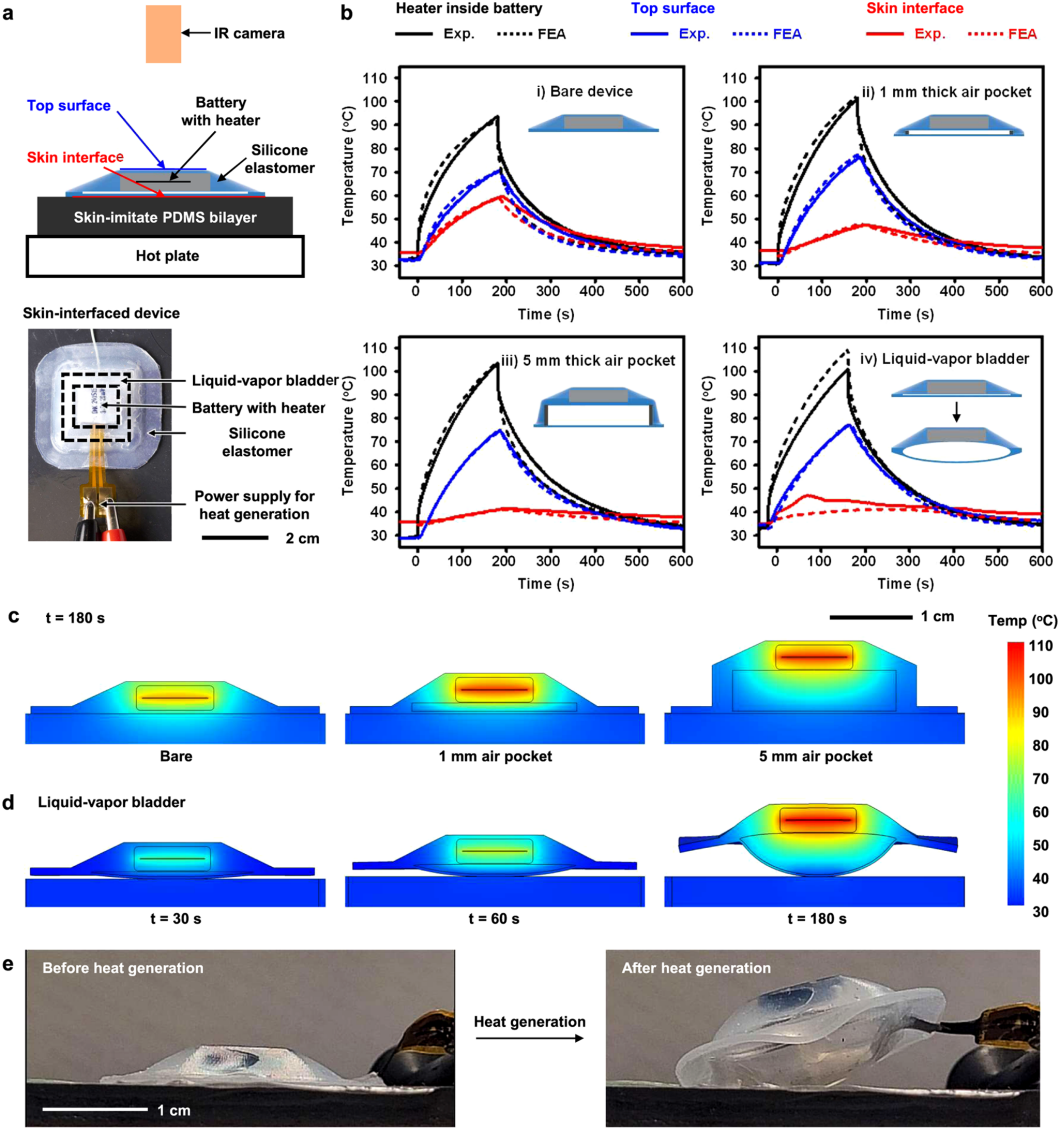
Operational features of a thermal safety system based on a liquid–vapor bladder. **a** Photographs of an experimental setup and a skin-interfaced device that includes a liquid–vapor bladder and a battery with heater (BwH) to simulate heat generation during thermal malfunction. **b** Representative temperature profiles of the heater inside the battery, the top surface of the device, and the skin interface for cases of a (i) bare device, (ii) 1 mm air pocket, (iii) 5 mm air pocket, and iv) liquid–vapor bladder. **c** Temperature distributions of the bare device, the device with 1 mm air pocket, and the device with 5 mm air pocket at 180 s after initiation of heating. **d** Thermomechanical deformation, delamination, and temperature distribution of the device with a liquid–vapor bladder at 30, 60, and 180 s. **e** The skin-interfaced device with liquid–vapor bladder before and after initiation of heating.

Heat from thermal runaway associated with battery failures represents the most significant risk for injury in many types of battery-powered skin-interfaced devices. Experiments described here simulate this process through Joule heating of a resistive element inserted into a battery pouch and connected to an external power supply. Figure 3a shows the experimental setup associated with this battery-with-a-heater (BwH) construct, for a device that rests on a skin phantom formed using a bilayer of poly(dimethyl siloxane) (PDMS) on a hot plate controlled to a temperature of 36.5 °C. The measured data consist of temperatures captured at the top surface of the device with an infrared (IR) camera, at a point inside the BwH, and at the skin interface with separate thermocouples. Details appear in the Methods and in Supplementary Figs. 11–13.

Experimental studies of thermal runaway use (i) a bare device, (ii) a device with 1 mm and (iii) 5 mm thick air pockets integrated under the BwH, as structures for thermal isolation, and (iv) a device with a liquid–vapor bladder under the BwH. The details are in Supplementary Fig. 14 and Methods. Figure 3b shows the temperature profiles that result from 250 mA of current applied to the heating element in the BwH for 180 s, to reach a temperature inside the BwH larger than 90 °C, slightly higher than that expected from rapid failure of a battery with a size typical of a wireless, skin-interfaced physiological monitor (lithium polymer battery, LiPol Battery, 45 mAh, Supplementary Fig. 15). In the case of the bare device, the temperature of the top surface reaches 70 ± 1 °C, and the skin interface reaches 59.8 ± 0.6 °C. For devices with 1 mm and 5 mm thick air pockets, the temperatures at the skin interface are 47.0 ± 0.2 °C and 41.0 ± 0.3 °C, respectively. The corresponding temperatures at the top surfaces of the BwH are 75.3 ± 0.9 °C and 76.2 ± 0.2 °C (Supplementary Fig. 16). Based on FEA results, at 180 s, the rates of heat transfer to the skin through the bottom of the bare device and the devices with 1 mm and 5 mm air pockets are 0.49 W (for total heat generation: 0.73 W), 0.41 W (0.75 W), and 0.18 W (0.75 W), respectively (Supplementary Table 1). As an important metric of the degree of thermal isolation from the skin, the corresponding heat transfer ratios to the skin, defined as the heat transfer rate to the skin / (the heat transfer rate to the upper air surroundings including the edges + the heat transfer rate to the skin) are 67, 55, and 24%. The temperature distributions, shown in Fig. 3c, are consistent with these trends. The air pocket structures provide effective thermal isolation, but they contribute to the overall thickness of the system.

By contrast, the liquid–vapor bladder creates a thermally isolating pocket only when needed, after temperatures exceed a threshold value (Fig. 3e, and Supplementary Movie 1). Experiments show that shortly after activating the BwH (0~15 s), the temperature at the skin-interface increases at a rate comparable to that with the bare device. When the liquid begins to evaporate (~15 s, ~41 °C, Supplementary Fig. 17b, and Supplementary Fig. 6), the bladder expands to form a thermally isolating pocket structure and, simultaneously, the evaporation process itself absorbs some of the heat, at 200 J/g. As a result of these combined effects, the rate of increase in the temperature at the skin interface sharply decreases (15~100 s, Supplementary Fig. 17a). Increasing the temperature above the boiling point expands the bladder, and the inflated bladder delaminates the device from the skin. The reduced heat transfer from the device and subsequent convective cooling start to decrease the skin temperature even during heat generation. This effect leads to a maximum temperature of the skin at only 44.8 ± 1.0 °C, well below the critical value of 48 °C for 1–10 min exposure (Supplementary Fig. 16). As in the example of Fig. 3b, the device with the heat source, BwH, delaminates from the substrate by a distance of ~2 mm due to bladder expansion at ~100 s, and ~46 °C (Supplementary Fig. 17c). The contact area of the device decreases to ~140 mm^2^ from ~1150 mm^2^ (~12%, Supplementary Fig. 17c). The bladder continues to expand (Supplementary Fig. 17d, e), and the resulting fully inflated bladder serves as a thermal barrier to maintain the temperature at the skin interface to values below 48 °C during 1 min to 10 min exposure. The device delaminates by a distance of 4.4 mm and 5.1 mm from the substrate, and the contact area decreases to 100 mm^2^ (~9%) and 14 mm^2^ (~1%) at ~200 s, and ~260 s, respectively. (Supplementary Fig. 17d, e). In fact, expansion of the liquid–vapor bladder decreases the heat transfer ratio to the skin from 29.3% at 30 s, 16.2% at 60 s, and 5.4% at 180 s (Supplementary Table 1). For example, if the device does not delaminate from the skin, FEA simulations yield corresponding ratios of 32.7% at 30 s, 26.9% at 60 s, and 29.7% at 180 s (Supplementary Table 1), comparable to the 5 mm air pocket device, as expected. The bladder remains in its expanded state after heating (Supplementary Fig. 17), thereby maintaining an effective thermal barrier indefinitely, for practical purposes, for temperatures above the boiling point of the liquid. These collective effects allow the liquid–vapor bladder to maintain a safe temperature at the skin interface even for demanding conditions, such as a total heat generation of nearly 1 W (0.93 W) for 10 min (Supplementary Fig. 18). Moreover, expansion of the bladder generates forces sufficient to induce delamination for the case of a device bonded to a PDMS substrate using a double-sided adhesive (3 M, 2477 P), to help maintain the skin interface temperature to values below the medical standard (Supplementary Fig. 19).

### Integration of the thermal safety system into a wireless, skin-interfaced health monitor

**Fig. 4:**
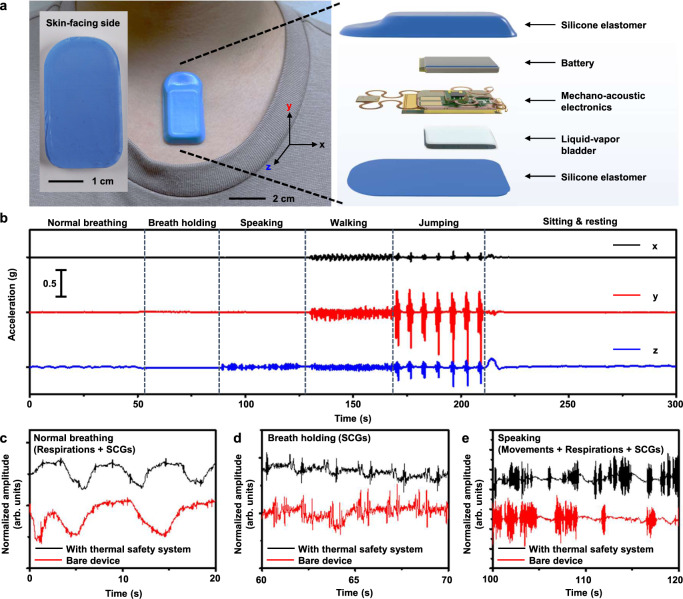
Operation of a skin-interfaced, wireless mechano-acoustic (MA) sensor with an integrated thermal safety system. **a** Photographs and an exploded schematic illustration of the MA device with a liquid–vapor bladder. The inset is a photograph of the skin-facing side of the device. **b** Three-axis accelerometry recorded with the device over 300 s during various activities, including normal breathing, breath holding, speaking, walking, jumping, sitting, and resting. **c**–**e** Respirations, cardiac activities (SCGs), and movements measured by the MA device during normal breathing, breath holding, and speaking, respectively, with thermal safety system (black, extracted from **b**, *z*-axis), and a bare device (red).

Practical demonstrations involve integration of this thermal safety system into the base of a skin-interfaced wireless sensor of mechanical and acoustic (i.e. mechano-acoustic) signatures of body processes^10^ (Fig. 4a). The device includes a high-bandwidth 3-axis accelerometer to monitor vibratory motions of the skin associated with vocalization and respiratory activity, cardiac cycles as well as changes in body orientation and physical activity. The bladder locates between the bottom surface of the flexible printed circuit board of the device and the base encapsulation layer. This addition increases the device thickness (~6 mm) by 0.125 mm and its weight (~5.8 g) by 40 mg. The overall flexibility and stretchability of the device remain unchanged because the bladder rests under the battery, which itself is rigid, and does not bend or stretch during use.

Figure 4b shows the representative data from a healthy adult subject. Normal breathing, breath holding, speaking, walking, jumping, and sitting and resting generate characteristic features in the time series data. Accelerations in the direction perpendicular to the surface of the skin (*z*-axis) contain strong signatures of respiratory and cardiac activity. As shown in Fig. 4c–d, respiratory cycles and the cardiac sounds, i.e. seismocardiograms (SCGs), appear clearly. Also, in Fig. 4e, speaking leads to high frequency features and associated harmonics. The presence of the thermal safety system has no measurable effect on the key features of the data, such as chest movement amplitude and signal-to-noise ratio (SNR) (Supplementary Fig. 20).

**Fig. 5:**
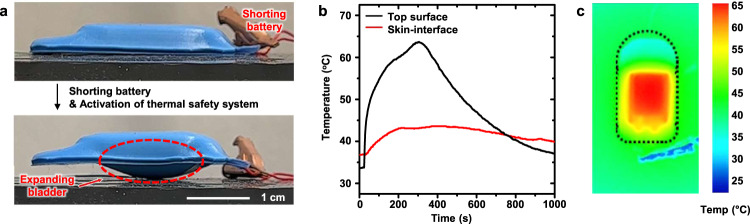
Activation of a thermal safety system in a skin-interfaced, wireless mechano-acoustic (MA) sensor. **a** Activation of the thermal safety system upon shorting the battery to cause catastrophic failure. **b** Temperature profile and **c** thermal map of the MA device.

Figure 5a shows the action of the thermal safety system that follows shorting of the actual battery in a device of this type, leading to a catastrophic failure. The resulting heat leads to expansion of the liquid–vapor bladder, thereby thermally isolating and physically delaminating the device from the skin phantom (Supplementary Movie 2). Figure 5b, c shows the temperature profiles and distributions. The temperature of the top surface of the device approaches 65 °C, but the temperature of the skin remains below 48 °C. Moreover, simulations of thermal failure of a battery in a device package that includes a thermal safety system, while mounted on the skin, lead to no visible redness, blisters or other signs of thermal damage to the skin (Supplementary Fig. 21).

## Discussion

This paper introduces a thin, lightweight bladder structure designed to protect the skin from thermal injury following certain types of malfunction in skin-integrated electronic devices. Upon overheating, this thermally activated system not only forms a highly effective thermal barrier between the skin and the device but it also simultaneously delaminates the device from the skin, as an additional form of protection. The automatic, self-powered operation of this materials-based design provides a fail-safe complement to standard electronic safety systems. Incorporation into the base encapsulating structure of a device adds this important function with negligible increases of cost, thickness, weight, size or mechanical rigidity, applicable across a wide-ranging scope of sensor types and configurations. As such, this technology has potential for widespread, practical use in both exploratory devices for research purposes as well as commercialized systems for patient care.

## Methods

### Fabrication of the liquid–vapor bladder

The enclosure consisted of a pair of films of EVOH (Non-catch, 50 μm, Kyodoprinting) manually cut to desired shapes and heat sealed on three sides (20 mm × 20 mm). Heat sealing of the fourth side followed injection of Novec 71DE liquid through this opening. A final cutting process removed excess parts of the EVOH films.

### Fabrication of the air pocket

A square hollow acrylic spacer sealed with polyester (PET) film on top and bottom surrounds the air inside. Laser cutting (CO_2_ laser cutter, ULS) of 1 mm and 5 mm of acrylic plates creates a hollow square (20 mm × 20 mm) column structure with an 800 μm thick wall. Bonding 150 μm thick PET films on the top and bottom of the column structure with glue completed the fabrication of the air pocket.

### Fabrication of the battery with heater

An ultraviolet laser cutter (Protolaser U4, LPKF) ablated the copper coating on a thin, flexible film (AP8535R, Pyralux, DuPont) of copper/PI/copper (thickness: 18 μm/75 μm/18 μm), to pattern metal interconnect traces with widths of 60 μm. Cutting the seal of the pouch of a lithium polymer battery allowed removal of the active components (DNK201515, DNK power) and attachment of a copper resistive heating element. Reinsertion into the pouch and resealing completed the fabrication of the battery with heater (BwH) device.

### Fabrication of the bilayer skin phantom

The skin phantom structures included two layers of silicone polymers to mimic the epidermis and the underlying soft tissue^22^. The latter (6 mm, thermal conductivity ~0.5 W/mK)^23,24^ was formed by mixing the base and curing agent of a commercial polymer kit (1:1, Sylgard 170, Dow Corning, the thermal conductivity of silicone made by Sylgard 170 is 0.48 W/mK)^25^, casting a sheet and curing at room temperature for 24 h. The former (100 μm, thermal conductivity 0.2 ~ 0.37 W/mK)^23,24^ was formed by mixing the base and curing agent of the same kit but at a different ratio (10:1, Sylgard 184, Dow Corning, the thermal conductivity of silicone made by Sylgard 184 is 0.27 W/mK)^26^, casting and curing at room temperature for 24 h. The casting process used a silicon wafer for the thin top film, an acrylic plate for the bottom film, and with spacers to control the thickness. Plasma treating the surfaces enabled a strong bond to form upon contact, to yield the final bilayer structure.

### Experimental setup for simulating thermal runaway

The experiments used skin-interfaced devices with integrated BwHs. Placing the skin phantom on a hot plate allowed its temperature to be controlled to values near those associated with skin. Thermocouples (k-type, Omega) placed at the surface of the phantom at a depth 1 mm below its surface allowed temperature recordings from these two locations with thermometer data loggers (HH374, Omega). Heat generated from inside the battery followed from application of constant current to the BwH through an external connection to a power supply (Keithley 2602 Source Meter, Tektronix). The voltage drop across the BwH (Digital multimeter, National Instruments) enabled a calculation of its internal temperature. An infrared (IR) camera (FLIR A600, Teledyne FLIR LLC) captured the temperature at the top surface of the device. A small current (0.5 mA) applied to the heater allowed continuous measurements of the temperature during cooling, after the heating process.

### Fabrication of the skin-interfaced device for thermal failure simulation (Fig. 3)

The devices used encapsulation structures (Supplementary Fig. 14) formed with soft elastomers, defined using computer-aided design software (SOLIDWORKS 2019, Dassault Systemes) and a corresponding pair of molds created in aluminum using a milling machine (MDX-540, Rolland DGA). Specifically, casting polyorganosiloxane on these molds and curing at 75 °C for 40 min created the top encapsulating structures (Supplementary Fig. 22a). Spin-coating at 250 rpm on an acrylic plate and curing at 75 °C for 40 min and patterning by laser cutter (CO_2_ laser cutter, ULS) yielded bottom encapsulation layers (Supplementary Fig. 22b). A liquid–vapor bladder or air pocket was bonded to the surface of the bottom layer, aligned with the center of the battery location, and the BwH was placed above the bladder. Casting uncured polyorganosiloxane elastomer (Ecoflex 00-30, Smooth-on) and curing at room temperature for 24 h after engaging the layers completed the fabrication. Curing process tightly bonded the top and bottom encapsulation layers by filling the space (Supplementary Fig. 22c).

### Fabrication of the skin-interfaced mechano-acoustic device (Fig. 4)

All other processes are the same with the fabrication of the skin-interfaced device for thermal failure simulation except for inserting electronics^10^ instead of the BwH.

### Finite element analysis

The commercial software COMSOL revealed aspects of heat transfer and thermal expansion of the skin-interfaced device on the surface of the skin phantom. Dynamic mechanical analysis (RSA-G2 Solids Analyzer, TA Instruments) determined the stress, strain, and temperature relationships for the EVOH films (Noncatch, Kyodoprinting) for use in the simulations under control of temperature from 20 to 100 °C (Supplementary Fig. 23). FEA simulated the thickness of the bladder as a function of the inner pressure load at various temperatures, which is summarized in Supplementary Fig. 24. The intrinsic property of the liquid–vapor saturation pressure at the controlled temperature is from the equation of the state of Novec 71DE. Thermophysical properties of Novec 71DE in liquid and vapor states were from REFPROP (Reference Fluid Thermodynamic and Transport Properties Database) by the National Institute of Standards and Technology (NIST), as shown in Supplementary Fig. 25. Effective in- and out-of-plane thermal conductivity of battery were extracted from temperatures of the heater and of battery surface as a function of the power applied to the heater (Supplementary Fig. 12). The effective heat capacity and the density are average properties weighted by the mass of electrodes and electrolytes^27^. Additional properties, including the other materials used for simulations, are summarized in Supplementary Table 2. A fully coupled heat transfer and mechanical deformation model captured the thermomechanical expansion of the liquid–vapor bladder triggered by heat. Thermal vaporization increased the pressure, which acted as a boundary load to induce bladder expansion. The mechanics of delamination from the skin was introduced into the FEA model. The first calculation defined the coupled deformation and delamination mechanics. A second calculation using a heat transfer model determined the temperature profile and inner pressure. After iterations with convergence, the decoupled models yielded mechanical deformation, delamination, and temperature profiles.

### Human subject studies

The feasibility of a thermal safety system with a skin-interfaced mechano-acoustic sensor was tested by a healthy adult subject under approval by the Northwestern University Institutional Review Board, Chicago, IL, USA (STU00214800). The subjects took part following informed consent.

### Statistical analysis

Thermomechanical expansion of the bladder was quantitatively performed with five samples (Fig. 2c). Numerical data were expressed with mean ± standard deviation, and the finite element analysis used the mean values. Heat generation simulations with BwH were independently performed with three samples for each condition and repeated with a similar result. Representative data (Fig. 3b) were from experiments, and the finite element analysis used the values from the representative data. The heat generation level of BwHs was characterized for each with a repeatability test (Supplementary Fig. 13), and reused for each condition to generate the same heat level.

### Reporting summary

Further information on research design is available in the Nature Portfolio Reporting Summary linked to this article.

## Supplementary information

Supplementary Information

Description of Additional Supplementary Files

Supplementary Movie 1

Supplementary Movie 2

Reporting Summary

## Data Availability

The data that support the findings of this study are available within the main text and the Supplementary Information. The data generated for the studies shown in Figs. 1–5 are provided in the Supplementary Information/Source data files. All data are also available from the corresponding authors upon request. Source data are provided with this paper.

## Source data

Source Data
