# Supplementary material for: Responsive materials and mechanisms as thermal safety systems for skin-interfaced electronic devices
Source: Nat Commun. 2023 Feb 23;14:1024. doi: 10.1038/s41467-023-36690-y (PMC9950147; doi:10.1038/s41467-023-36690-y)
Supplement: Supplementary file 1 — Supplementary Information [file 41467_2023_36690_MOESM1_ESM.pdf]

Supplementary Information

Responsive Materials and Mechanisms as Thermal Safety Systems for Skin-Interfaced Electronic Devices

Seonggwang Yoo[1†], Tianyu Yang[1,2†], Minsu Park[1], Hyoyoung Jeong[1,3], Young Joong Lee[1], Donghwi Cho[1,4], Joohee Kim[1,5], Sung Soo Kwak[1,5], Jaeho Shin[1], Yoonseok Park[6], Yue Wang[1,7], Nenad Miljkovic[2], William P. King[2*], John A. Rogers[1,7,8,9,10*]

[1]Querrey Simpson Institute for Bioelectronics, Northwestern University, Evanston, IL 60208, USA

[2]Department of Mechanical Science and Engineering, University of Illinois at Urbana-Champaign, Urbana, IL 61801, USA

[3]Department of Electrical and Computer Engineering, University of California, Davis, CA 95616, USA

[4]Thin Film Materials Research Center, Korea Research Institute of Chemical Technology, Daejeon 34114, Republic of Korea

[5]Center for Bionics of Biomedical Research Institute, Korea Institute of Science and Technology, Seoul 02792, Republic of Korea

[6]Department of Advanced Materials Engineering for Information and Electronics, Kyung Hee University, Yongin 17104, Republic of Korea.

[7]Department of Biomedical Engineering, Northwestern University, Evanston, IL 60208, USA

[8]Department of Materials Science and Engineering, Northwestern University, Evanston, IL 60208, USA

[9]Department of Chemistry, Northwestern University, Evanston, IL 60208, USA

[10]Department of Neurological Surgery, Feinberg School of Medicine, Northwestern University, Chicago, IL 60611, USA

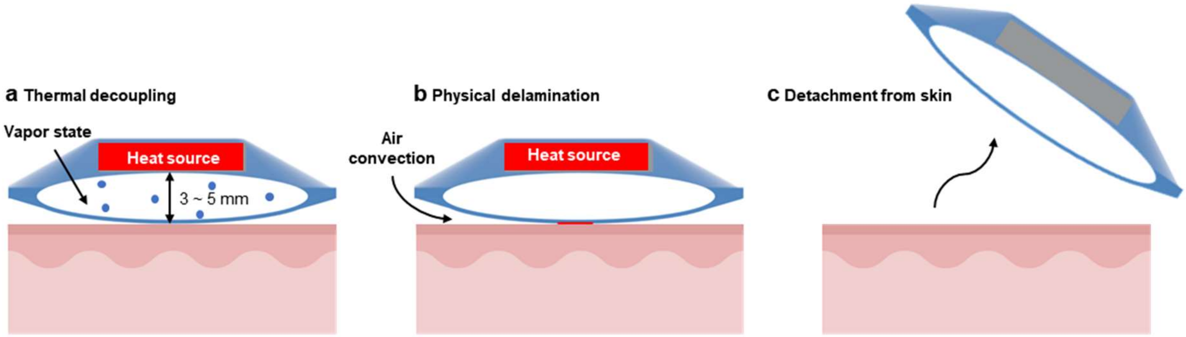

Supplementary Fig. 1 | Illustrations of mechanisms initiated in the thermal safety system by thermal malfunction of a device. **a** Thermal decoupling, **b** physical delamination, and **c** detachment from the skin.

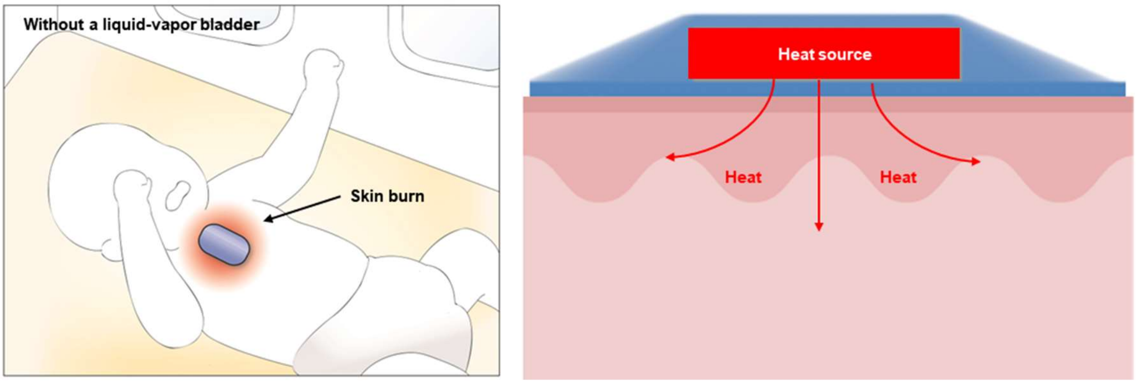

Supplementary Fig. 2 | Illustrations of thermal malfunction of a device while on the skin.

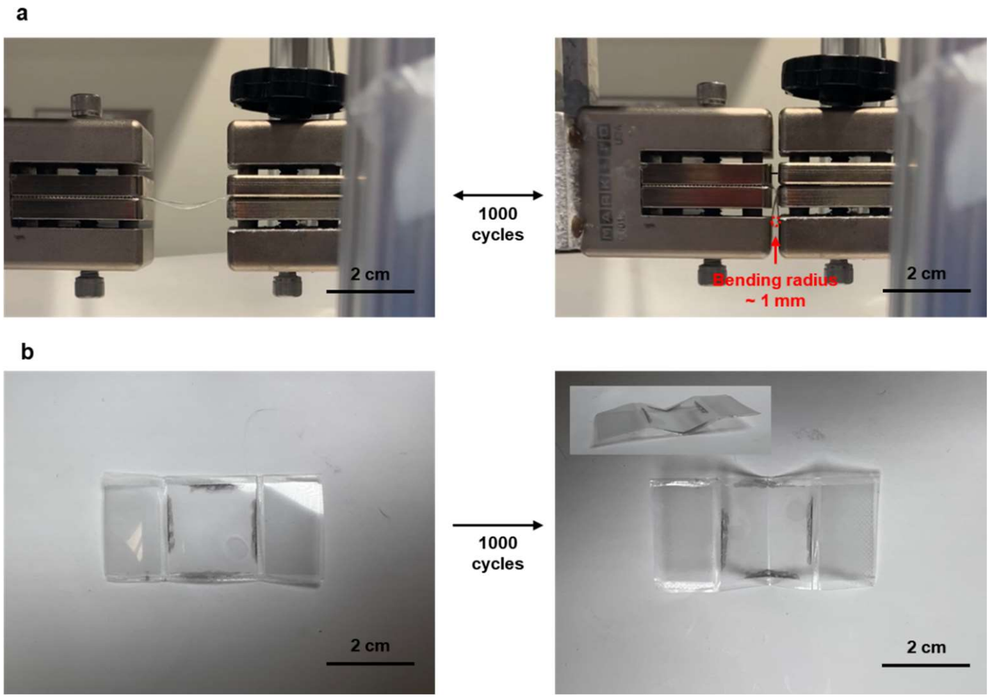

Supplementary Fig. 3 | Cyclic bending test of the liquid-vapor bladder (Bending radius: ~1 mm, 1000 cycles). a Photographs of the experimental set up for cyclic bending tests, and **b** liquid-vapor bladder before and after test.

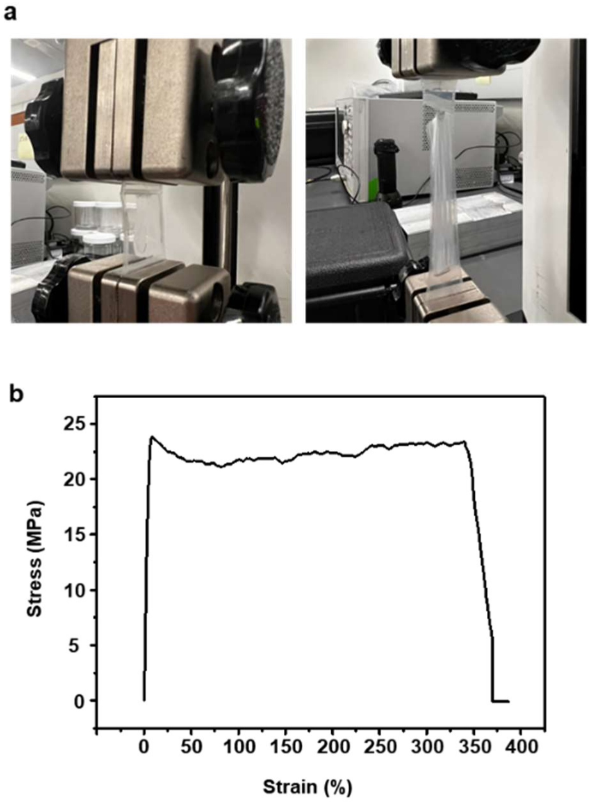

Supplementary Fig. 4 | Tensile tests of the liquid-vapor bladder. a Photographs of the experimental set up for tensile tests of the liquid-vapor bladder, and **b** strain-stress curve of the bladder. The fracture strain of the sealed bladder is ~340%.

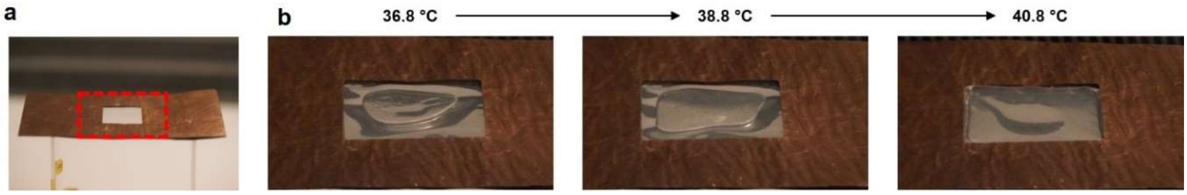

Supplementary Fig. 5 | Experimental set up to illustrate the thermomechanical expansion of liquid-vapor bladder. a Angled view photographs, and **b** vaporization of liquids inside the bladder.

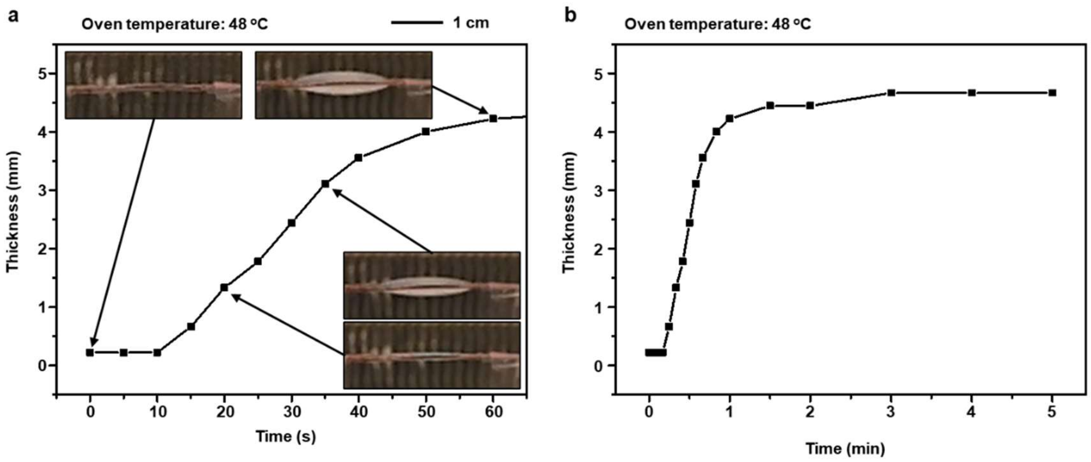

Supplementary Fig. 6 | a,**b** Thermomechanical expansion of a liquid-vapor bladder in an oven at 48 °C.

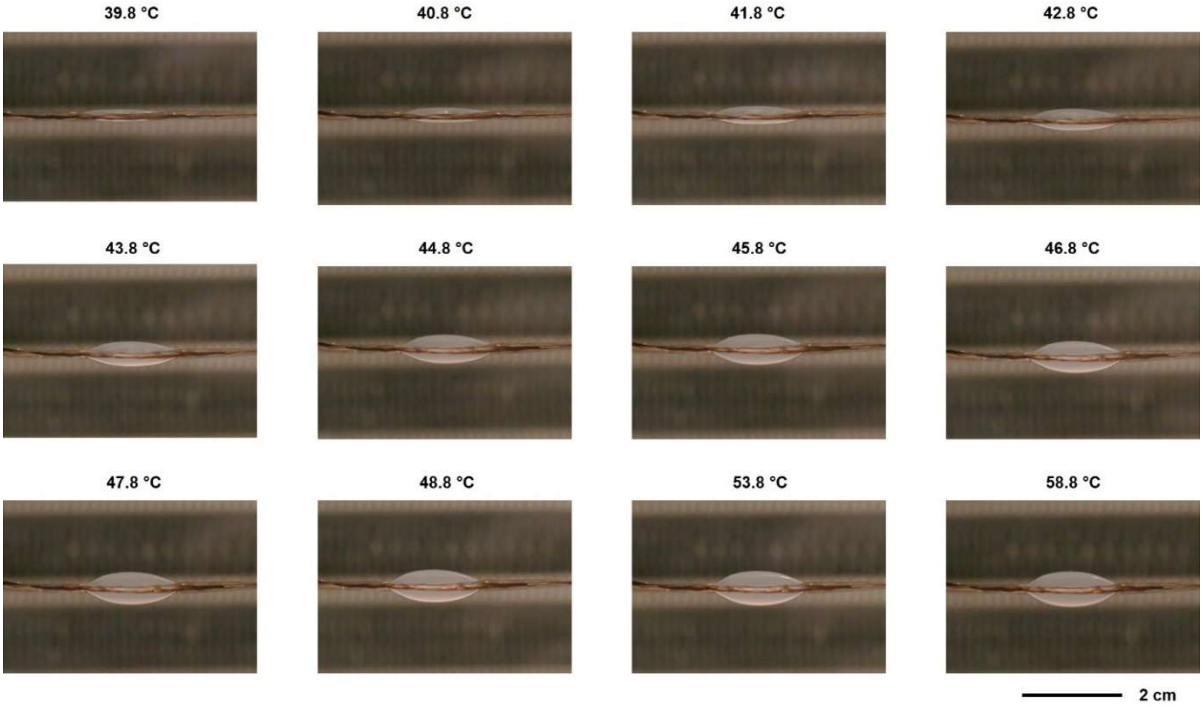

Supplementary Fig. 7 | Representative photographs of a liquid-vapor bladder captured at various temperatures. The amount of liquid is ~10 µL.

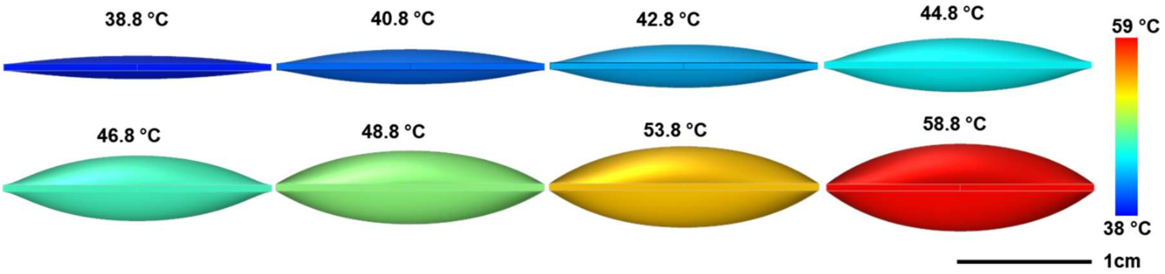

Supplementary Fig. 8 | Simulated thermomechanical deformations and temperature distributions of the liquid-vapor bladder at various temperatures. The liquid is depleted at 46.6 °C.

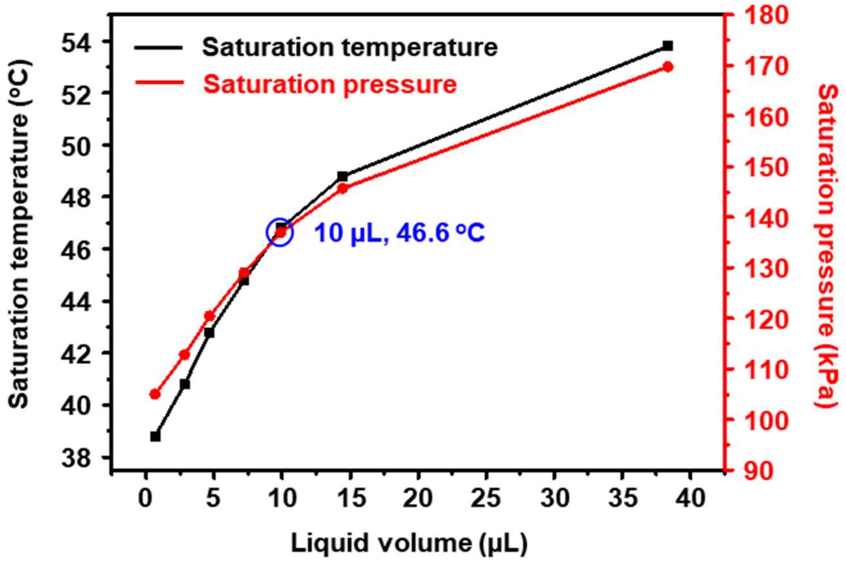

Supplementary Fig. 9 | Saturation temperature (boiling point) and saturation pressure of Novec 71DE liquid inside the 20 mm × 20 mm EVOH bladder with various volumes.

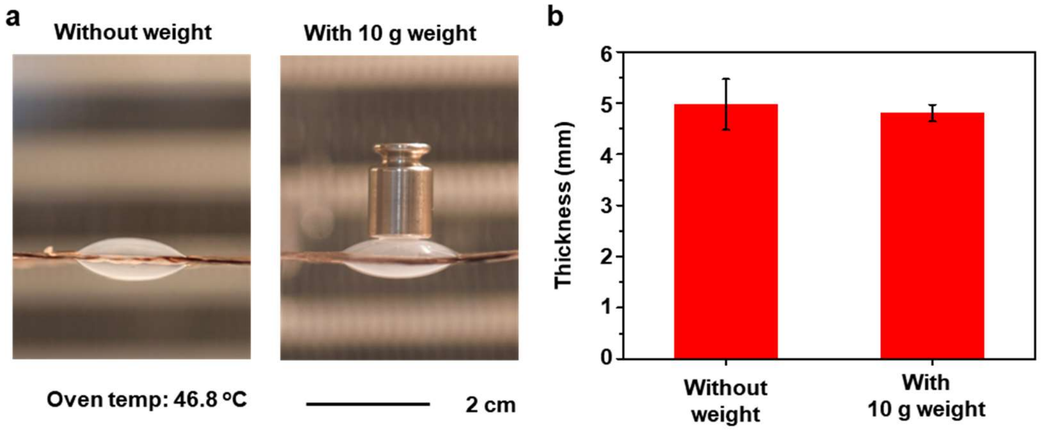

Supplementary Fig. 10 | Thermomechanical expansion of a liquid-vapor bladder with and without a 10 g weight placed on top. a Photographs without and with a 10 g weight, and **b** the maximum thickness following expansion for these two cases.

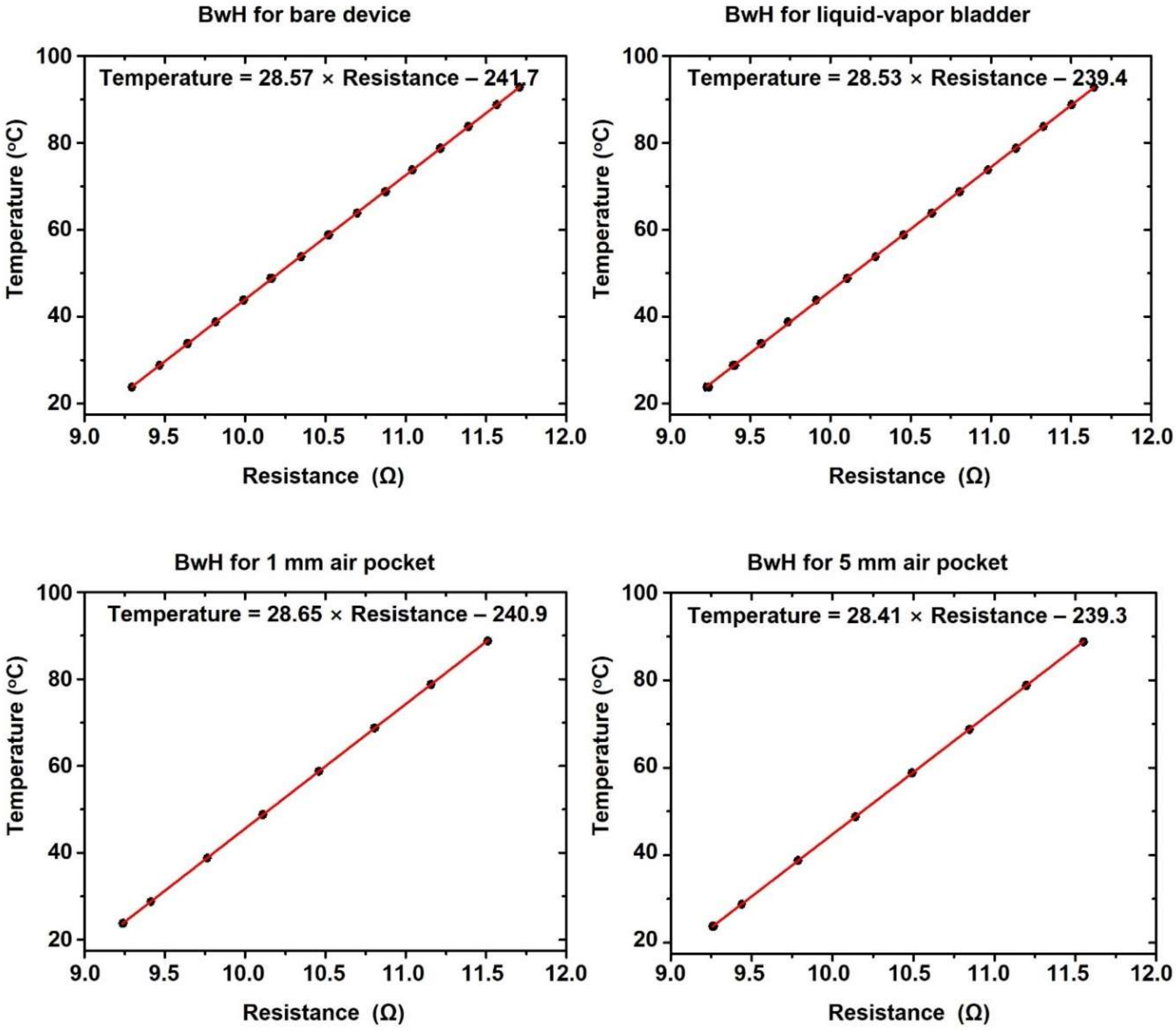

Supplementary Fig. 11 | Temperature of the BwH as a function of resistance of the heater, as calibration for determining the temperature inside the BwH during testing of the thermal safety system.

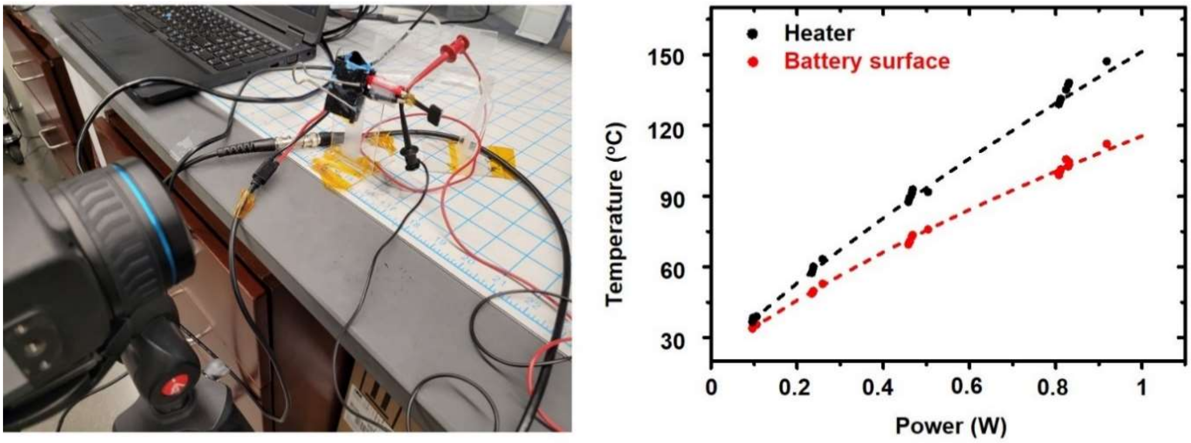

Supplementary Fig. 12 | Experimental set up and heat generation by the BwH as a function of power.

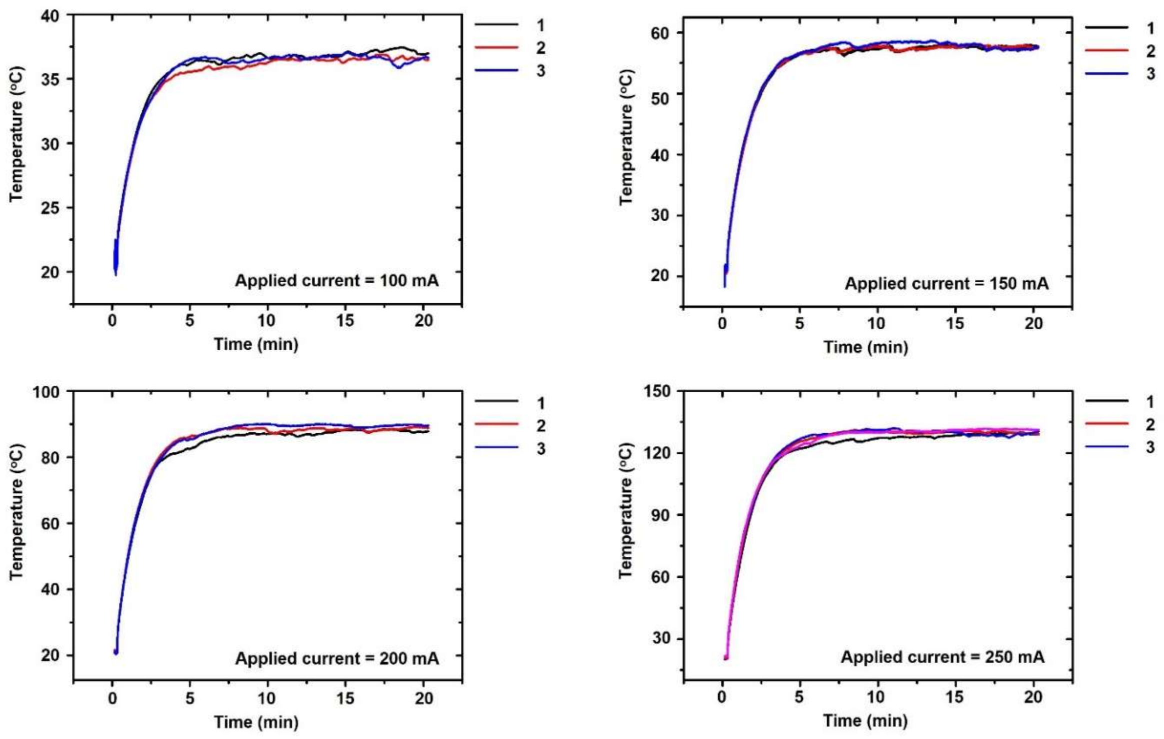

Supplementary Fig. 13 | Plots of temperature as a function of time after initiating heating in a BwH with different applied currents, as tests of repeatability.

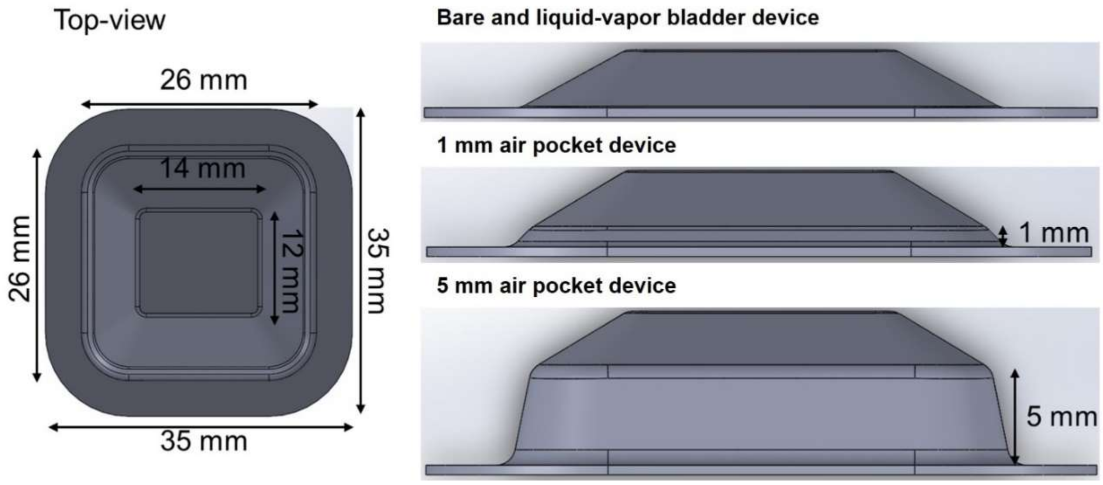

Supplementary Fig. 14 | Top-view and side-view of a bare device, a device with liquid-vapor bladder, and with 1 mm and 5 mm air pockets.

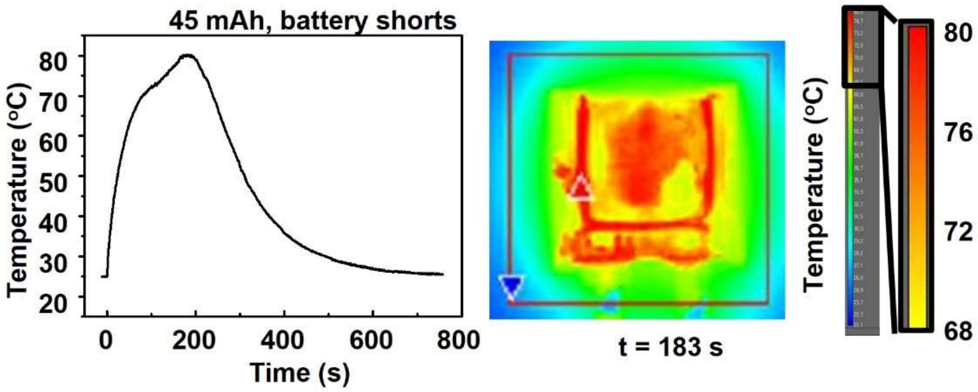

Supplementary Fig. 15 | Temperature profile and temperature map of a battery (lithium polymer battery, LiPol Battery, 45 mAh) during an electrical short and associated catastrophic failure.

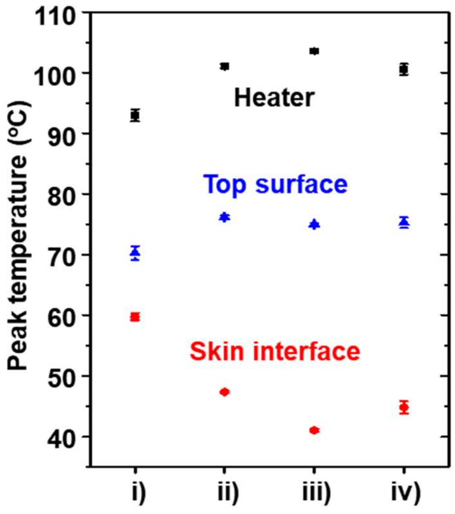

Supplementary Fig. 16 | Peak temperature of heater inside the battery, the top surface of the device, and the skin interface for cases of a i) bare device, ii) 1 mm air pocket, iii) 5 mm air pocket, and iv) liquid-vapor bladder.

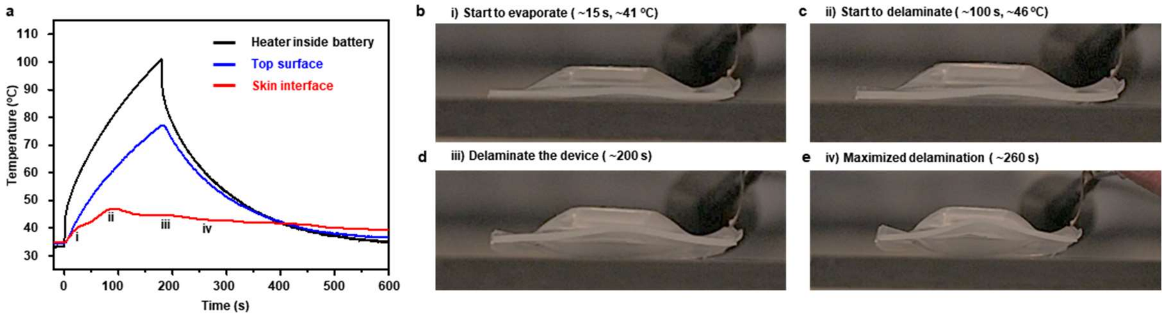

Supplementary Fig. 17 | Simulations of thermal failure of a battery in a device package that includes a liquid-vapor bladder. a Temperature profiles and (**b-d**) photographs of the device at different times during heat generation.

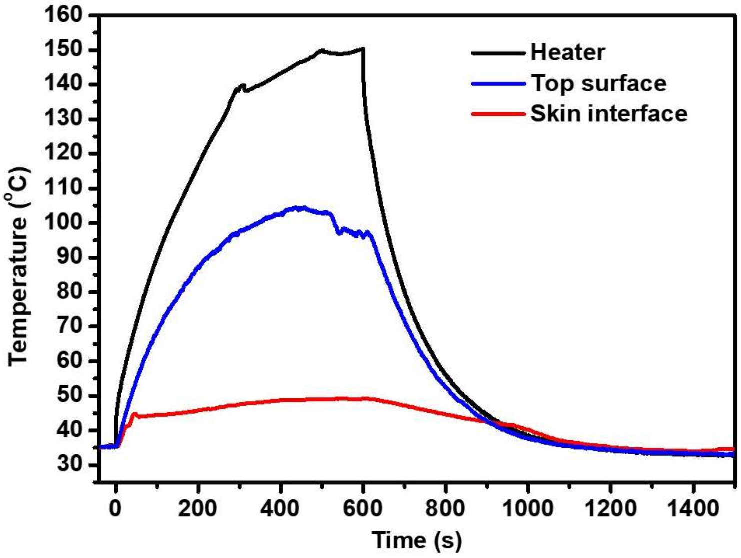

Supplementary Fig. 18 | Temperature profiles of the heater inside the battery, the top surface of the device, and the skin interface with a liquid-vapor bladder for the case of 0.93 W of thermal power applied for 600 s.

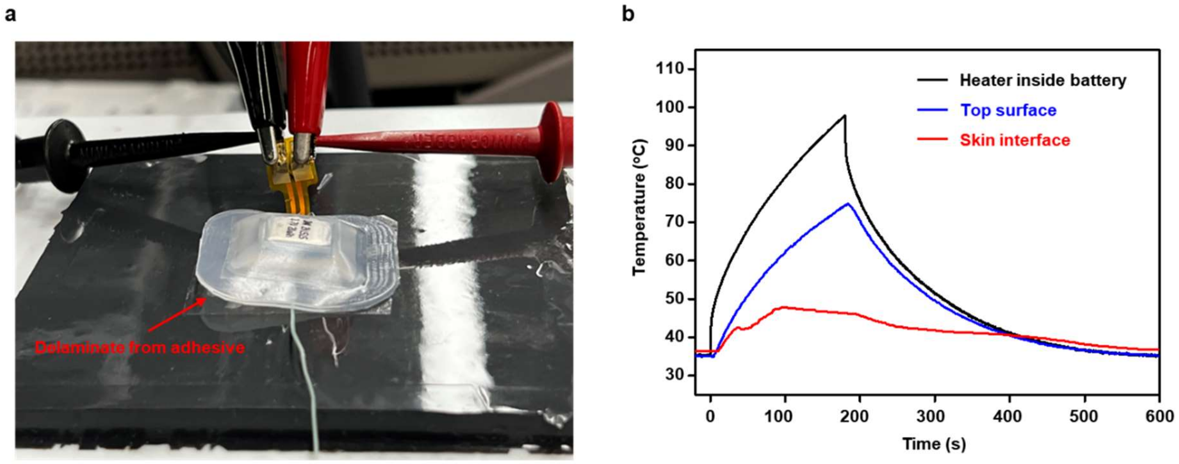

Supplementary Fig. 19 | Activation of a thermal safety system during thermal failure simulation while strongly bonded to a substrate of PDMS using a medical adhesive. a Photograph of a device after expansion of the bladder and associated delamination from the substrate. **b** Temperature profiles during the simulation.

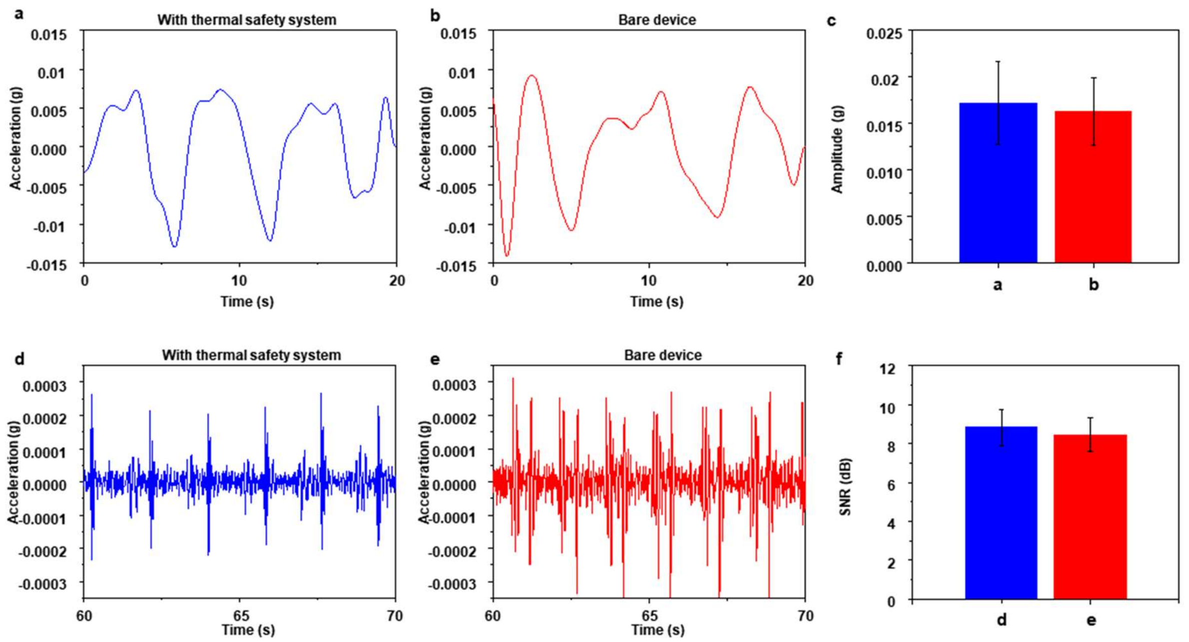

Supplementary Fig. 20 | Comparison of the features of the data in Fig. 4. a,**b** Bandpass filtered data from Fig. 4c (Filtering frequency: 0.1-1 Hz), and **c** amplitude of chest movements. **d**,**e** Bandpass filtered data from Fig. 4d (Filtering frequency: 10-40 Hz), and **f** signal-to-noise ratio (SNR).

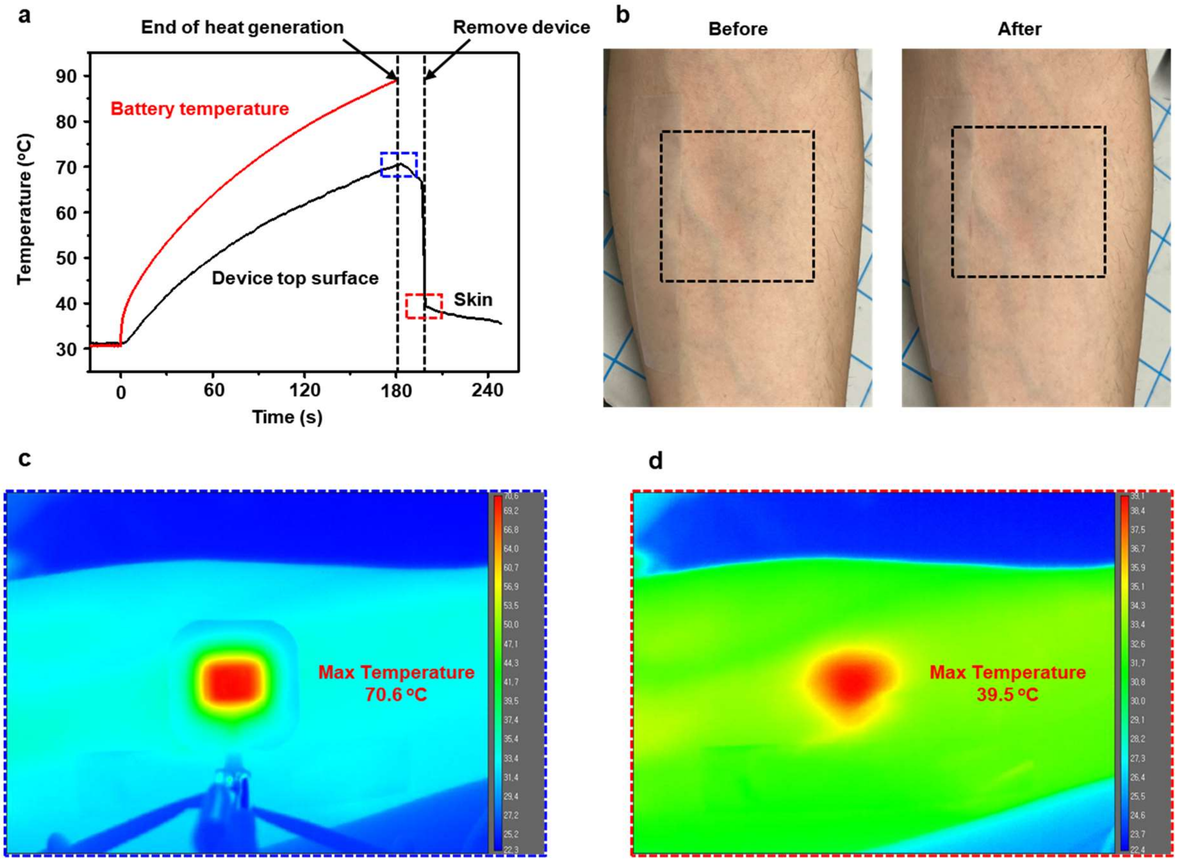

Supplementary Fig. 21 | Studies of the skin interface during simulations of thermal failure of a battery in a device package that includes a liquid-vapor bladder. **a** Temperature profiles during thermal failure simulation. **b** Photographs of skin at the location of the device before application and after removal following a thermal failure simulation. **c, d** IR images corresponding to times indicated by the **c** blue and **d** red boxes in the graph in **a.**

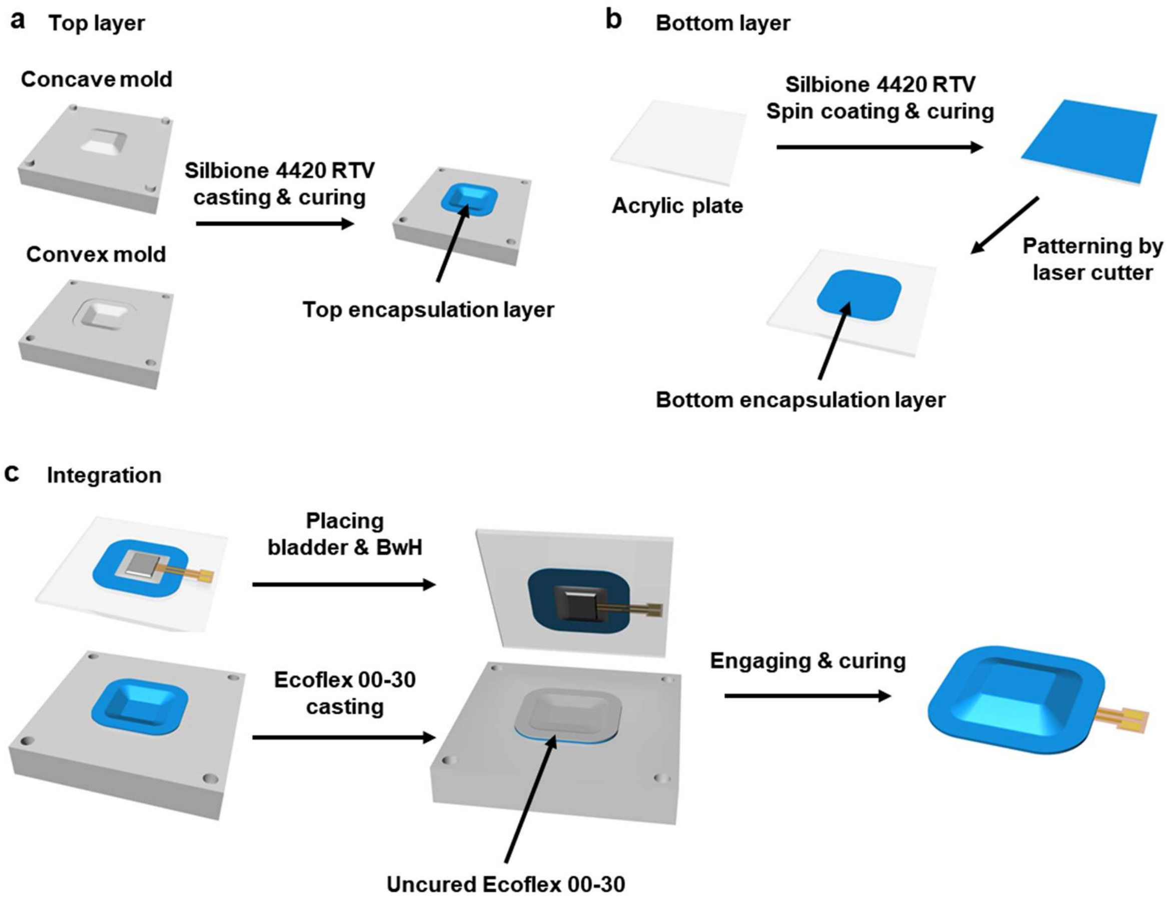

Supplementary Fig. 22 | Schematic illustration of the device fabrication process. Process for **a** top encapsulation layer, **b** bottom encapsulation layer, and **c** integration of the device.

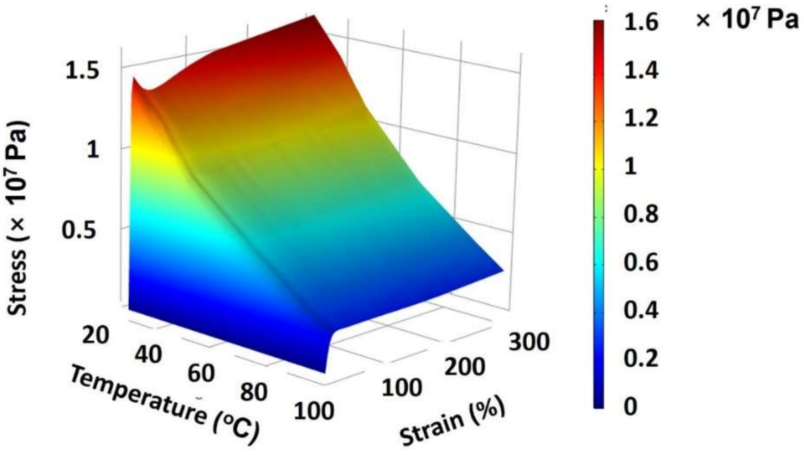

Supplementary Fig. 23 | Stress, strain, and temperature behavior of a liquid-vapor bladder. The strain-stress curve is obtained at each temperature by using DMA.

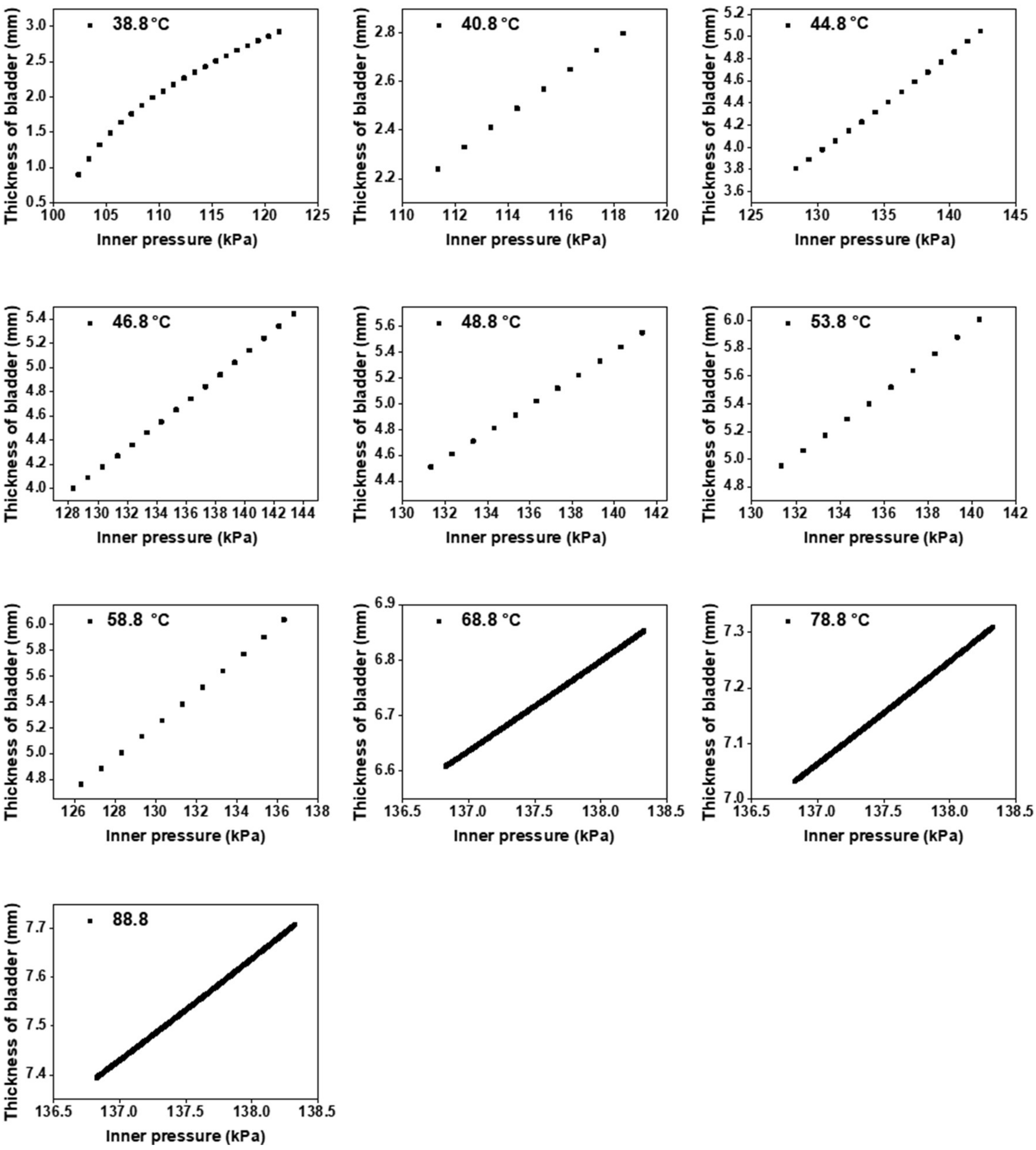

Supplementary Fig. 24 | FEA simulation of the thickness of the bladder as a function of the inner pressure at various temperatures.

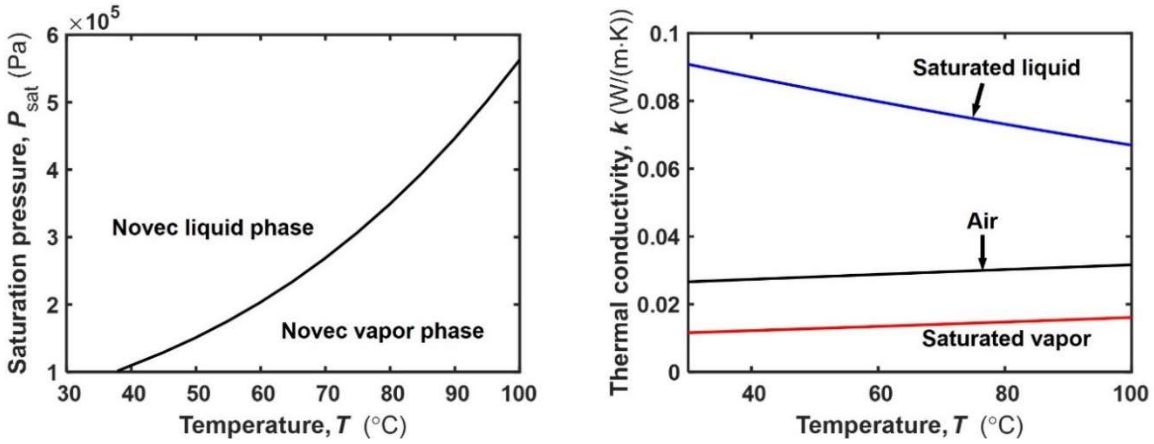

Supplementary Fig. 25 | Saturation pressure of Novec 71DE in liquid and vapor phases and the thermal conductivity of the saturated liquid, vapor, and air as a function of temperature.

Supplementary Table 1 | Total heat generation and heat transfer rates through the top and bottom surfaces of the device for each case.

Case	Total heat generation (W)	Top heat transfer rate to air (W)	Bottom heat transfer rate to skin (W)
i (bare device at 180 s)	0.73	0.24	0.49
ii (1mm air pocket at 180 s)	0.75	0.34	0.41
iii (5mm air pocket at 180 s)	0.75	0.57	0.18
iv (liquid-vapor bladder at 30 s)*	0.66	0.14	0.064
iv (liquid-vapor bladder at 60 s)*	0.68	0.33	0.058
iv (liquid-vapor bladder at 180 s)	0.74	0.7	0.04
iv, with no delamination (liquid-vapor bladder at 30 s)*	0.66	0.14	0.068
iv, with no delamination (liquid-vapor bladder at 60 s)*	0.68	0.25	0.092
iv, with no delamination (liquid-vapor bladder at 180 s)	0.74	0.52	0.22

*For highly transient heat transfer at 30 s and 60 s, majority of the heat generation is absorbed by temperature increases of battery, vaporization of liquid-vapor bladder, and silicone materials.

Supplementary Table 2 | Thermal and mechanical properties of materials used in thermal simulations.

Properties	Thermal conductivity, k (W/(m·K))	Heat capacity, Cp (J/(kg·K))	Density, ρ (kg/m^3)	Young's modulus, E (MPa)	Poisson's ratio, ν
Skin	0.37	3391	1109	0.1	0.48
Battery[S1]	1.0 (in plane)<br>0.14 (out of plane)	1760	535	1000	0.35
Sylgard170 phantom	0.43	1703	1370	1.76	0.4
Ecoflex 00-30	0.25	1460	1080	3.5	0.4
Air	0.024	700	1.2	-	-

Reference

S1. Villano, P., Carewska, M., Passerini, S. Specific heat capacity of lithium polymer battery components, *Thermochim. Acta* **402**, 219-224 (2003).
