# Supplementary material for: Responsive materials and mechanisms as thermal safety systems for skin-interfaced electronic devices
Source: Nat Commun. 2023 Feb 23;14:1024. doi: 10.1038/s41467-023-36690-y (PMC9950147; doi:10.1038/s41467-023-36690-y)
Supplement: Supplementary file 2 — Description of Additional Supplementary Files [file 41467_2023_36690_MOESM2_ESM.pdf]

Description of Additional Supplementary Files

Supplementary Movie 1: Activation of the thermal safety system with a thermal failure simulation.

Supplementary Movie 2: Activation of the thermal safety system with a shorting of the battery inside the mechano-acoustic skin-interfaced device.
