# Supplementary material for: Responsive materials and mechanisms as thermal safety systems for skin-interfaced electronic devices
Source: Nat Commun. 2023 Feb 23;14:1024. doi: 10.1038/s41467-023-36690-y (PMC9950147; doi:10.1038/s41467-023-36690-y)
Supplement: Supplementary file 5 — Reporting Summary [file 41467_2023_36690_MOESM5_ESM.pdf]

nature portfolio

Corresponding author(s): William P. King, John A. Rogers

Last updated by author(s): Jan 26, 2023

Reporting Summary

Nature Portfolio wishes to improve the reproducibility of the work that we publish. This form provides structure for consistency and transparency in reporting. For further information on Nature Portfolio policies, see our Editorial Policies and the Editorial Policy Checklist.

Statistics

For all statistical analyses, confirm that the following items are present in the figure legend, table legend, main text, or Methods section.

n/a	Confirmed	
☐	☒	The exact sample size (n) for each experimental group/condition, given as a discrete number and unit of measurement
☐	☒	A statement on whether measurements were taken from distinct samples or whether the same sample was measured repeatedly
☒	☐	The statistical test(s) used AND whether they are one- or two-sided<br>*Only common tests should be described solely by name; describe more complex techniques in the Methods section.*
☒	☐	A description of all covariates tested
☐	☒	A description of any assumptions or corrections, such as tests of normality and adjustment for multiple comparisons
☐	☒	A full description of the statistical parameters including central tendency (e.g. means) or other basic estimates (e.g. regression coefficient) AND variation (e.g. standard deviation) or associated estimates of uncertainty (e.g. confidence intervals)
☒	☐	For null hypothesis testing, the test statistic (e.g. F, t, r) with confidence intervals, effect sizes, degrees of freedom and P value noted<br>*Give P values as exact values whenever suitable.*
☒	☐	For Bayesian analysis, information on the choice of priors and Markov chain Monte Carlo settings
☒	☐	For hierarchical and complex designs, identification of the appropriate level for tests and full reporting of outcomes
☒	☐	Estimates of effect sizes (e.g. Cohen's d, Pearson's r), indicating how they were calculated

Our web collection on statistics for biologists contains articles on many of the points above.

Software and code

Policy information about availability of computer code

Data collection	Microsoft Excel<br>Thermometer data logger (HH374, Omega) with thermocouples (k-type, Omega)<br>Infrared (IR) camera (FLIR A600, Teledyne FLIR LLC)<br>Digital multimeter (National Instruments)
Data analysis	OriginPro<br>FLIR ResearchIR

For manuscripts utilizing custom algorithms or software that are central to the research but not yet described in published literature, software must be made available to editors and reviewers. We strongly encourage code deposition in a community repository (e.g. GitHub). See the Nature Portfolio guidelines for submitting code & software for further information.

Data

Policy information about availability of data

All manuscripts must include a data availability statement. This statement should provide the following information, where applicable:

- Accession codes, unique identifiers, or web links for publicly available datasets
- A description of any restrictions on data availability
- For clinical datasets or third party data, please ensure that the statement adheres to our policy

The data that support the findings of this study are available within the main text and the Supplementary Information. The data generated for the studies shown in Figure 1-5 are provided in the Supplementary Information/Source data files. All data are also available from the corresponding authors upon reasonable request.

Human research participants

Policy information about studies involving human research participants and Sex and Gender in Research.

Reporting on sex and gender	2 male, Sex and gender of participants are not relevant to our study.
Population characteristics	Age: 27, 30
Recruitment	The healthy adult subjects are participate to demonstrate the feasibility of a thermal safety system with a skin-interfaced mechano-acoustic sensor. The selection of the patient do not impact on result to our study.
Ethics oversight	The demonstration of a feasibility of a thermal safety system with a skin-interfaced mechano-acoustic sensor was tested by healthy adult subjects under approval by the Northwestern University Institutional Review Board, Chicago, IL, USA (STU00214800). The subjects took part following informed consent.

Note that full information on the approval of the study protocol must also be provided in the manuscript.

Field-specific reporting

Please select the one below that is the best fit for your research. If you are not sure, read the appropriate sections before making your selection.

☒ Life sciences ☐ Behavioural & social sciences ☐ Ecological, evolutionary & environmental sciences

For a reference copy of the document with all sections, see nature.com/documents/nr-reporting-summary-flat.pdf

Life sciences study design

All studies must disclose on these points even when the disclosure is negative.

Sample size	No sample-size calculations were performed, because the goal of this study was to demonstrate the feasibility of a thermal safety system with a skin-interfaced mechano-acoustic sensor.
Data exclusions	No data were excluded.
Replication	All experiments were performed repeatedly, and the thermomechanical behavior of the bladder and the device was repeated.
Randomization	Allocation was not random and not relevant to our study.
Blinding	Blinding is not relevant to our study for the demonstration of the feasibility of a thermal safety system with a skin-interfaced mechano-acoustic sensor.

Reporting for specific materials, systems and methods

We require information from authors about some types of materials, experimental systems and methods used in many studies. Here, indicate whether each material, system or method listed is relevant to your study. If you are not sure if a list item applies to your research, read the appropriate section before selecting a response.

Materials & experimental systems

n/a	Involved in the study
☒	☐ Antibodies
☒	☐ Eukaryotic cell lines
☒	☐ Palaeontology and archaeology
☒	☐ Animals and other organisms
☒	☐ Clinical data
☒	☐ Dual use research of concern

Methods

n/a	Involved in the study
☒	☐ ChIP-seq
☒	☐ Flow cytometry
☒	☐ MRI-based neuroimaging
